# Formation Mechanism and Cohesive Energy Analysis of Metal-Coated Graphene Nanocomposites Using In-Situ Co-Reduction Method

**DOI:** 10.3390/ma11112071

**Published:** 2018-10-23

**Authors:** Yuanlin Xue, Wenge Chen, Jiaojiao Wang, Longlong Dong, Qian Zhao, Yongqing Fu

**Affiliations:** 1 School of Materials Science and Engineering, Xi’an University of Technology, Xi’an 710048, China; xueyuanlinxyl@163.com (Y.X.); wjjxaut@126.com (J.W.); zqtomorrow0017@163.com (Q.Z.); 2 Advanced Materials Research Central, Northwest Institute for Nonferrous Metal Research, Xi’an 710016, China; 3 Faculty of Engineering and Environment, Northumbria University, Newcastle upon Tyne NE1 8ST, UK

**Keywords:** graphene, coated metals, preparation, characterization, mechanisms

## Abstract

Nanocomposite powders based on metal-coated graphene were synthesized using an in-situ co-reduction method in order to improve wettability and interfacial bonding between graphene and metal. Graphene oxide (GO) of 2~3 atomic layers was synthesized using the Hummer’s method with graphite as a raw material and then dispersed into a dispersing agent solution mixed with *N*-Methyl pyrrolidone and deionized water to form a homogeneous GO suspension, which was finally added into electroless plating solutions for the reduction process. Copper-coated graphene (Cu@graphene) and nickel-coated graphene (Ni@graphene) were synthesized using this one-step and co-reduction method by mixing salt solutions containing metal ions and GOs into the plating solution. The Cu ions or Ni ions were adsorbed and bonded onto the edges and surfaces of graphene, which was reduced from the GOs using a strong reducing agent of ascorbic acid or sodium borohydride. Crystalline Cu particles with an average size of about 200 nm were formed on the surface of graphene, whereas amorphous or nanocrystalline Ni particles with an average size of 55 nm were formed on the surface of graphene. Distribution of these metal particles on the graphene is homogeneous and highly dispersed, which can effectively improve the sinterability of composite powders. Cohesive energy distribution between graphene and metal interface was analyzed using first-principle calculation method. Formation mechanism of metal coated graphene was identified to be that both the GO and metal ions were simultaneously reduced in the reducing agents and thus a chemical bonding of graphene/metal was formed between the metal particles and graphene.

## 1. Introduction

Graphene has excellent physical and mechanical properties owing to its unique structure [1]. In fact, graphene of 2~3 atomic layers shows good electrical performance [2]; graphene of 6~7 atomic layers exhibits a remarkable thermal characteristic [3]; and graphene of about 10 atomic layers possesses outstanding mechanical properties [4]. These excellent properties make graphene one of the most promising materials for wide-range applications, including nano-electronics, composite materials, catalysts, sensors, energy storage, transistors, touch screens and gene sequencing and so forth [5,6,7,8,9].

Graphene is commonly used as a reinforcing material in composites, including metal, polymer and ceramic matrix composites [10,11,12,13,14]. However, its difficulty of uniform dispersion into the matrix, poor wettability to many substrate materials and poor interfacial bonding with matrix seriously restrict its successful applications in composite materials. One of the critical issues is how to improve its interfacial bonding with the metal matrix in order to efficiently transfer the applied load and enhance electron/heat transport. It is critical to find solutions to uniformly disperse the graphene nanoflakes as an efficient reinforce agent and avoid their agglomeration.

An efficient method was proposed to pre-coat the surface of graphene with a layer of metal in order to prevent its agglomeration in the matrix [15,16]. Due to the improved dispersion and good interfacial bonding, thermal energy and electrons can be effectively transferred between graphene and the coated metal and thus the wettability of metal-coated graphene can be significantly improved. The so-formed metal coated graphene composite powders (in this paper we will use metal@graphene) will maintain good properties of both graphene and metal and could find wide-range applications in the fields of energy, catalysis, biomedicine, sensors and spectroscopy. There are extensive studies to synthesize various types of metal@graphene composites, using solvothermal method, cation exchange, thermal exfoliation, chemical vapor deposition and so forth [17,18,19,20,21]. Hassan et al. [22] prepared uniform and well-dispersed metallic nanoparticles on chemically converted graphene sheets using a chemical reduction method assisted by microwave irradiation. Wang et al. [23] fabricated Ni–graphene nanocomposites using a multi-step electroless deposition and Ni nanoparticles with an average diameter of 20 nm were decorated onto reduced graphene oxide (rGO) sheets. Kuang et al. [24] prepared Ni/graphene composites using an electro-deposition with GO sheets mixed inside a Ni sulfamate solution and graphene in the composites was reduced from the GO sheets during an electro-deposition process. Cao et al. [25] synthesized Ni/graphene nanocomposites using a chemical vapor deposition approach. As reported in the literature, the commonly used methods for preparing metal@graphene are focused mainly on chemical synthesis methods, however, these are often multi-step and high cost processes.

Therefore, in the present work, we synthesized Cu@graphene and Ni@graphene using a new and efficient in-situ co-reduction method. The formation mechanism of the metal@graphene composite was analyzed based on various characterization results. The cohesive energy between metal and graphene was calculated and analyzed using the first principle calculation method.

## 2. Materials and Methods

### 2.1. Raw Materials

In this work, the raw materials used were flakes of graphite powders (200 mesh, 99.90% purity, Nanjing Xian Feng Nano Materials Technology Co. Ltd., Jiangsu, China). Chemical reagents, such as copper sulfate, nickel sulfate, sodium hydroxide, concentrated sulfuric acid (98%), HCl (37%), H_2_O_2_ (30%), *N*-methyl pyrrolidone, NaOH (PH = 11), ammonia (25–28%), KMnO_4_, were bought from Sinopharm Chemical Reagent Co. Ltd., Shanghai, China. All the chemicals were used directly without further purification.

### 2.2. Fabrication of Graphene Oxide (GO)

The standard Hummer’s method was employed to synthesize GO [21,26,27]. A typical experiment can be summarized as follows: (a) 5 g of graphite powder was mixed with 115 mL concentrated H_2_SO_4_ and then stirred using an ice-bath (at 0 °C) for 30 min; (b) At room temperature of 25 °C, 20 g of KMnO_4_ was slowly added into the above mixture and agitated for 120 min; (c) The temperature of above mixture solution was increased to 35 °C during the process; and then 50 mL of deionized water (DI) was gradually added into the above solution; (d) 80 mL of DI water was gradually added into the system; After all the reactions were completed, hydrogen peroxide (30 wt %) was added into the solution until it turned into a yellow color and no bubbles were further produced; (e) The mixture was filtered and washed using HCl (5 wt %) and DI water for ~28 times and then dried at 70 °C for 12 h using a vacuum freeze-drying method. In this paper, we did not perform the recycling of the residual product as reported in Reference [28] but this will be explored in the future work.

### 2.3. Fabrication of Metal-Coated Graphene (Metal@Graphene) Nanocomposites Powders

**Figure 1:**
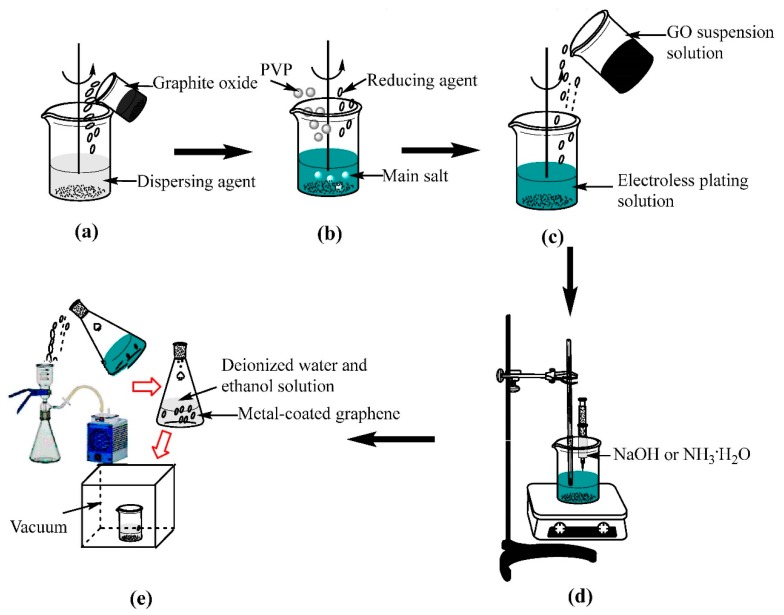
Illustration of the preparation process of Cu@graphene powders and Ni@graphene: (**a**) dispersion of oxidized graphite in dispersing agent; (**b**) electroless plating solution; (**c**) adding GO suspension solution to electroless planting solution and stirred; (**d**) mixture solution was stirred at the same time with dripped NaOH or NH_3_·H_2_O; (**e**) precipitates were filtrated, cleaned and freeze-dried.

As illustrated in Figure 1, metal@graphene composites powders were prepared using a thermo-electroless plating method. The compositions of the metal plating solutions (Cu or Ni) and reaction conditions are listed in Table 1.

The detailed electroless plating processes are listed as follows:

(a) 20 mg of GO was dispersed into 100 mL dispersing agent solution in an ultrasound bath for 3 h to obtain a homogeneous GO suspension solution (0.2 mg/mL) as shown in Figure 1a. The dispersing agent in the Cu plating and Ni plating are *N*-Methyl pyrrolidone and DI mixture solution;
(b) The salts (e.g., copper sulfate was used for copper plating and nickel sulfate for nickel plating), reducing agents (e.g., ascorbic acid in the copper plating and sodium borohydride in the nickel plating) and surfactant agents were mixed together to form an electroless plating solution (Figure 1b);
(c) Then the GO suspension solution was added into the electroless plating solution (Figure 1c);
(d) The mixed solution was intensively stirred using a magnetic stirrer to disperse the GO suspension solution. The copper plating process was done at 90 °C for 3 h and the nickel plating was done at 30 °C for 30 min. Simultaneously the NaOH solution or ammonia solution was dripped into the mixed solution to adjust the pH value to between 9 and 10 (Figure 1d). The pH value in the bath was continuously measured using a pH meter and was maintained by adjusting the added NaOH solution or ammonia solution using a peristaltic pump. The temperature during the electroless plating process was controlled using a fixed-temperature water bath.
(e) The precipitates were separated from the solution after the chemical reactions were completed (Figure 1e). DI water and ethanol solution were used to wash the precipitates for several times and dried at 60 °C for 8 h using a vacuum freeze-drying method.

### 2.4. Characterization

An X-ray diffractometer (XRD-7000S, Shimadzu, Japan) with Cu K_α_ radiation at 40 kV and 15 mA was used to analyze the crystalline structures of GO and metal@graphene. The scanning rate was 8°/min and the scanning range of 2θ was 5~65° with a step size of 0.02°. Surface morphology of the GO and metal@graphene was observed using a scanning electron microscope (SEM, TESCAN VEGA3 XMU, TESCAN, Brno, Czech Republic) and chemical element analysis was performed using an energy dispersive X-ray spectrometer (EDS, TESCAN, Brno, Czech Republic). Detailed morphological characteristics of the GO and metal@graphene composites were obtained using a transmission electron microscope (TEM, JEM-3010, JEOL, Akishima-shi, Japan). Characterization of samples using Raman spectroscopy (Via Reflex, Renishaw, London, Uk) were performed using a laser beam with a wavelength of 532 nm and a SWIFT detector over a range of 500–3500 cm^−1^ and all the spectra were taken at room temperature (20 °C). Fourier transform infrared (FT-IR) spectra of the GO and metal@graphene were obtained using a TENSOR 27 spectrophotometer (Bruker, Karlsruhe, Germany) with wavelengths ranging from 500 to 4000 cm^−1^ at room temperature. Microscale surface morphologies of metal@graphene powders were obtained using an atomic force microscope (AFM, FastScan, Bruker, Karlsruhe, Germany) in a tapping mode.

### 2.5. Modeling and Calculation Details

For the graphene system after atomic adsorption, there are defects, grain boundaries and intrinsic atoms formed. The adsorption energy of defects on the graphene is maximum and those of the grain boundary and intrinsic atoms on the graphene are approximately equal. The binding between graphene and metal is generally weaker than a typical covalent bond. In the present study, we focused on analysis of cohesive energies of Cu and Ni atoms with both their (111) surfaces bonded onto a graphene of a few atomic layers. The modeling work only considered the intrinsic atoms and defects adsorption. The first principle calculation was used to evaluate the cohesive energy between graphene and metal interface, as the binding force cannot be obtained using experimental methods easily.

**Figure 2:**
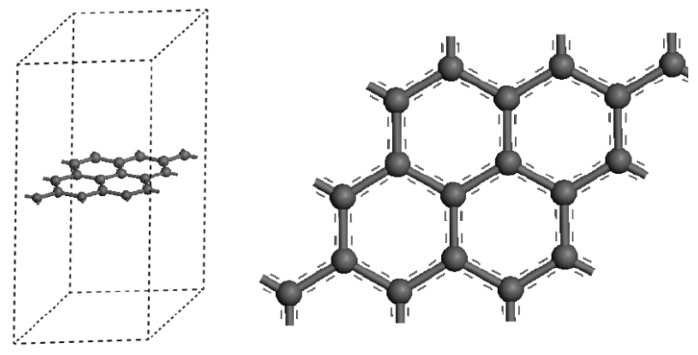
The model diagram of supercell of Ni or Cu coated graphene.

The detailed model information is described as follows: firstly, the lattice is established. The lattice constant is set as a = b = 2.46, c = 3.4 and carbon (related to graphene) atoms are added (a = 0.333, b = 0.667, c = 0.5). Then, in order to eliminate the influence of molecular bond between graphene layers, the vacuum layer is expanded to 2 nm on the Z axis direction. Finally, a and b in the supercell are increased to 3 times of original values and the initial configuration of the model is obtained (in Figure 2). The structures and properties of the system were investigated based on the density functional theory (DFT) using the Vienna Ab-initio Simulation Package (VASP) software and the DMol3 quantization package [29,30]. The self-consistent solution of the DFT equation was acquired by numerical integrations in order to obtain the electron property, wave function and charge distribution of the system. During the calculation, the settings were verified in order to achieve a total energy convergence less than 500 eV. For structural optimization, all the atoms are relaxed until all the atomic forces are less than 0.001 eV, also, the Brillouin zone is sampled by a 4 × 4 × 1of *k* points for the Monkhorst–Packs.

## 3. Results and Discussions

### 3.1. Microstructure Characterization and Morphology Analysis

**Figure 3:**
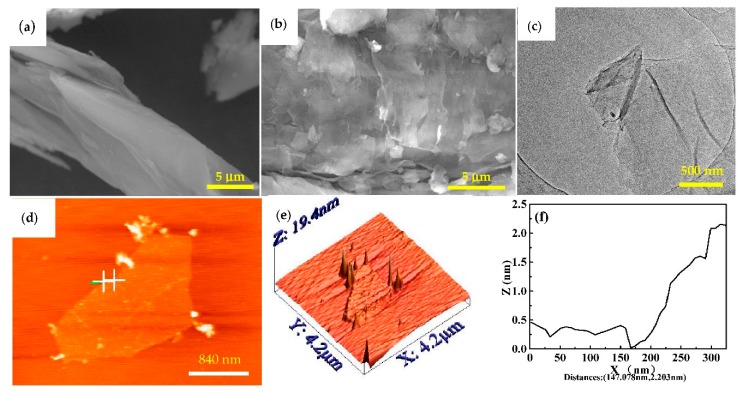
Scanning electron microscopy (SEM) images of (**a**) Flake graphite and (**b**) Graphene oxide (GO); (**c**) Transmission electron microscopy (TEM) and (**d**) Atomic Force microscopy (AFM) images of GO; (**e**) 3D image of surface image; (**f**) cross-section analysis of the height of graphene using AFM.

Figure 3a,b show SEM images of the flakes of graphite and GO. The graphite (Figure 3a) shows large sizes of flakes and its surface is smooth and flat. Whereas the surface of GO (Figure 3b) has many ripples, folds and bumps and is quite rough with apparent lamellar structures. The edges of the GO sheets have apparent folding structures. Comparison of the images in Figure 3a,b reveals that after the graphite was oxidized, the flat surface of the graphite disappears due to the oxidation process. There are various structural defects such as mosaic, vacancies and impurity atoms in the crystalline structure of graphite [31].

In order to check the thickness and microstructure of the GO, TEM and AFM images were obtained and the results are shown in Figure 3c–f. GO shows a crumpled sheet structure (Figure 3c). As can be seen from Figure 3d,e, the thickness of the GO sheet is about 2.203 nm. It was reported that the thickness of a monolayer GO was about 1.2 nm [32]. The thickness of the GO sheet in this study is thicker, which could be explained by the fact that the oxidized graphene sheets are bonded with hydroxyl and epoxy groups from both sides. Besides, the surface wrinkles and the presence of water molecules on the surface of the GO could also increase the measured thickness of the GO sheet from the AFM test [32]. The thickness of GO measured in this experiment was 2.203 nm, thus the thickness of GO could be about 2 to 3 layers.

**Figure 4:**
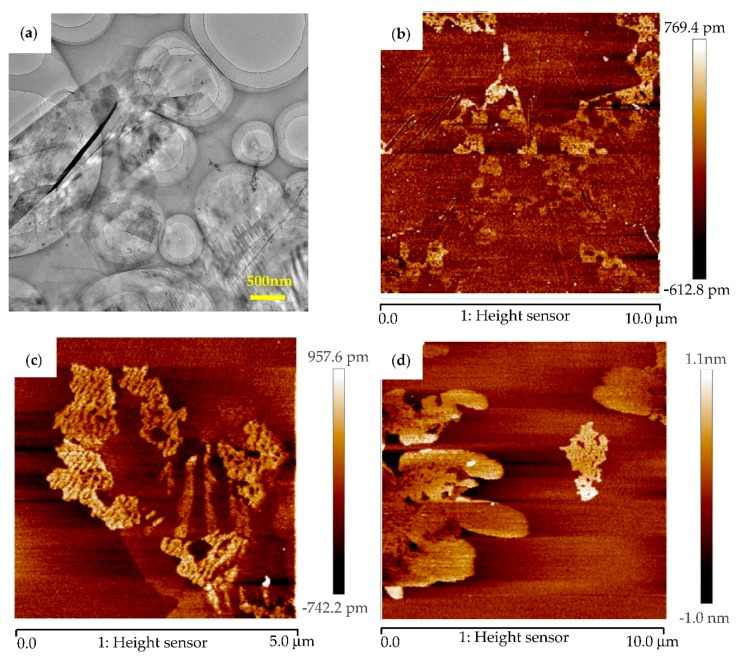
(**a**) TEM of graphene and AFM images of (**b**) graphene; (**c**) Cu-decorated graphene and (**d**) Ni-decorated graphene.

AFM and TEM images of graphene, Cu@graphene and Ni@graphene obtained from the in-situ co-reduction processes are shown in Figure 4. As shown in Figure 4a, the graphene is transparent with wrinkled structures. Comparing Figure 4b with Figure 4d, the Cu or Ni particles were coated on the surface of graphene which forms the loose and porous surfaces [33], suggesting that the thickness of the graphene after coated with the Cu or Ni particles would be increased.

**Figure 5:**
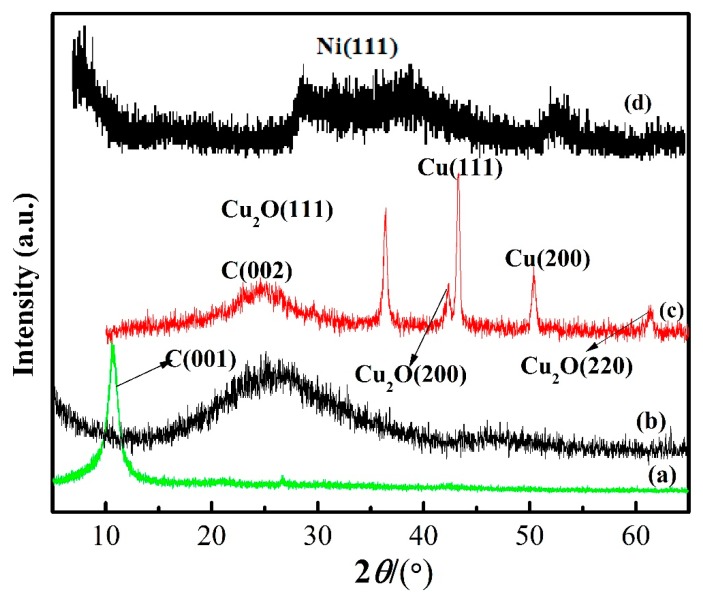
X-ray diffraction (XRD) patterns of (**a**) GO; (**b**) Graphene; (**c**) Cu@graphene and (**d**) Ni@graphene.

The obtained XRD patterns of GO sheets, graphene, Cu@graphene sheets and Ni@graphene sheets are shown in Figure 5. For the GO (Figure 5a), a sharp peak was observed near the diffraction angle 2θ of 10°, which is the characteristic peak of the GO [24]. This shows that the graphite has been successfully oxidized into GO through the intercalation process. Generally, the spacing of GO inter-planar spacing can be estimated using the Bragg Equation (1):

(1) 2dsinθ=nλ

where *n* is 1, *λ* is the X-ray wavelength of 0.154 nm from the Cu X-ray source, θ is half of the diffraction angle and d is the interlayer spacing. The calculated layer spacing of peak (001) of graphite oxide is ~0.80 nm, which indicates that the GO obtained is nearly a monolayer [34].

For XRD result of Cu@graphene (Figure 5c), there are three dominant diffraction peaks at 43.3°, 50.4° and 74.1°, respectively, corresponding to three face-centered cubic copper crystal (111), (200) and (220). The characteristic peak corresponding to the (002) crystal plane of graphene was found to be at 2θ = 26° and it is relatively weak when compared to the copper peaks [27]. The peaks of cuprous oxide were also found in the XRD pattern of Cu@graphene sheet, indicating that some cuprous oxides were formed during the chemical reaction process.

For XRD result of the Ni@graphene sheets (Figure 5d), there is a broad peak at 2θ = 44.5° which is corresponding to the (111) plane of nickel, indicating that the crystallinity of nickel on the surface of graphene is relatively poor and the nickel on the surface of graphene may be in an amorphous or nano-crystalline structure. However, there is no obvious peak observed near the diffraction angle 2θ of 26.3°, which is because the surface of graphene sheets is covered with Ni metal layer.

In order to verify that the graphene was still maintained after the in-situ co-reduction process, XRD analysis of the prepared composites (Figure 5b) shows a broad diffraction peak at 22.5°. Comparing Figure 5c with Figure 5d, it can be seen that the diffraction peak of the composite is similar to the graphene, instead of GO [35].

**Figure 6:**
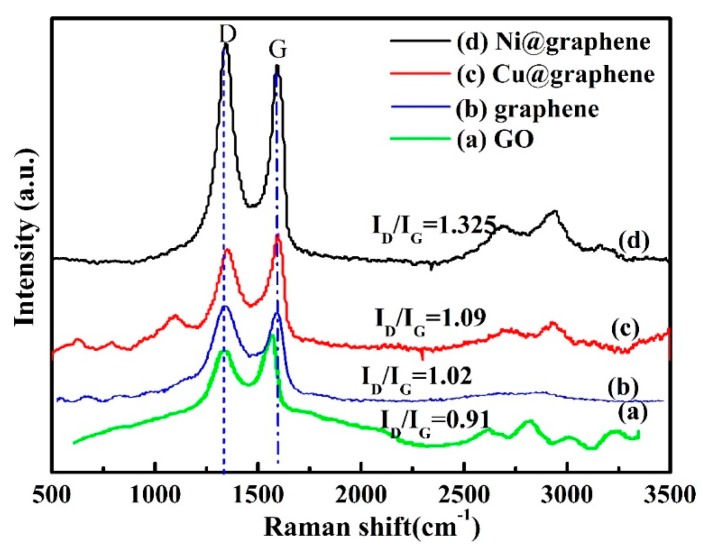
Raman spectra of (**a**) GO; (**b**) graphene; (**c**) Cu@graphene and (**d**) Ni@graphene.

Raman spectra of GO, graphene, Cu@graphene and Ni@graphene powders are presented in Figure 6. The Raman spectrum of the GO in Figure 6a exhibits three characteristic peaks at 1349.09, 1593.06 and 2700 cm^−1^, which represent the D peak, G peak and 2D peak of the GO. The intensity ratio of D peak and G peak (I_D_/I_G_) is often used to characterize the degree of defects in carbon materials [21]. The I_D_/I_G_ of GO in this study was measured to be 0.91 and it is similar to those reported in references [36,37].

Comparing the I_D_/I_G_ data of composites and graphite, we can confirm that the defects are increased after the graphite was oxidized and some of the carbon atoms in the defect structures are combined with the oxygen-containing functional groups [38]. Formation of these highly active sites of defects can promote the adsorption of metal ions in the subsequent plating process. After the graphite is oxidized, some of the carbon atoms in the structure are combined with the oxygen-containing functional groups and transform from *sp*^2^ hybridization into *sp*^3^ hybridization. The oxygen-containing functional groups not only cause changes in crystallinity of hexagonal network structures of the graphite but also increase the defects, which causes the G peak to be widened and the strength of D peak to be increased [39]. Whereas for graphene, the ratio of I_D_/I_G_ is 1.02 as shown in Figure 6b. Therefore, the increased ratio of I_D_/I_G_ for the composite indicates that majority of oxygen-containing functional groups were removed during the reduction process, the reason of which has been reported in our previous work [40,41].

Raman spectrum (Figure 6c) of the Cu@graphene composite exhibits two characteristic peaks: that is, ~1352.07 cm^−1^ and ~1593.06 cm^−1^, which represent the D peak and G peak of carbon. The calculated I_D_/I_G_ values of GO and Cu@graphene are 0.91 and 1.09, respectively. This indicates that the reduction effect will result in the increases of defects, topological disorders and the degree of graphitization; as well as decrease of crystallinity. The Raman spectrum of the Ni@graphene composite (Figure 6d) has two prominent peaks corresponding to ~1340.17 cm^−1^ of the D peak and ~1593.06 cm^−1^ of the G peak. It can be seen that the I_D_/I_G_ of Ni@graphene is 1.325, which is much larger than the value of 0.91 for the GO. This shows that after the surface of graphene is coated with nickel, the crystallinity is decreased and the defects are increased. This also shows that after the GO has been reduced into graphene, the oxygen-containing functional groups are decreased on the surface of GO [42]. Therefore, the π bond has been recovered and the defects and disorder are increased.

The Raman 2D peak (2711.88 cm^−1^) is originated from two phonon and double resonances of laser induced vibration process for carbon. Generally, the 2D peak is an indication if the graphene is single layer or a few layers [43]. Previous studies showed that the 2D peak was shifted to the direction of large wave-number side as the number of graphene layers was increased [36]. The unique 2D peak of the single-layer graphene is located at 2678.8 cm^−1^, whereas that of double-layer graphene is located at 2692.3 cm^−1^ and that of graphene having more than 10 layers is close to the position of the 2D peak of natural graphite which is located at 2716.5 cm^−1^. The 2D peak of GO in this study (Figure 6a) is located at 2700 cm^−1^, indicating the GO has a few layers [38]. The 2D peak of the Cu@graphene (Figure 6c) is located at 2706 cm^−1^, which indicates the layer of graphene is between two and ten. The 2D peak of the Ni@graphene (Figure 6d) is located at 2680 cm^−1^, which shows that the nickel-plated graphene has one monolayer. It also suggests that the graphene layer became thinner when the sodium borohydride was used as the reducing agent. This indicate that the graphene layer was slightly thicker when the ascorbic acid was used as the reducing agent.

**Figure 7:**
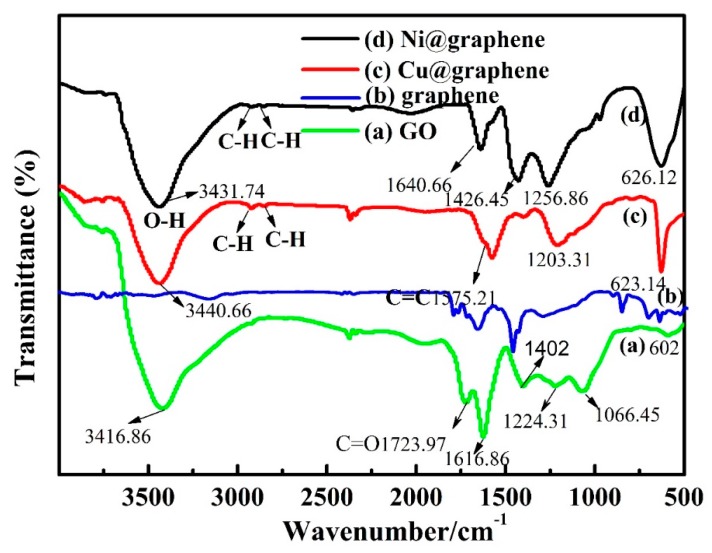
The Fourier transform-Infrared (FT-IR) spectra of (**a**) GO; (**b**) graphene; (**c**) Cu@graphene and (**d**) Ni@graphene.

Figure 7 shows the FT-IR spectra of the prepared samples. The FT-IR spectrum of the GO sheets in Figure 7a shows many peaks: for example, absorption peaks due to the –O–H stretching vibration of hydroxyl groups; water in the GO at 3416.86 cm^−1^; the –C=O stretching mode at 1723.97 cm^−1^; the vibration mode of –O–H at 1402 cm^−1^; the –C–OH stretching vibration at 1066.45 cm^−1^; and the absorption peak at 1616.86 cm^−1^ [44]. Based on the appearance of oxygen functional groups on the surface of GO measured by the FT-IR and the changes in the morphology of GO from the SEM observation, we can conclude that graphite has been intercalated into GO sheets. In general, the FT-IR spectrum of graphene is quite similar to that of GO [18]. However, the intensities of all the peaks correlated to the oxygen functional groups of graphene are decreased dramatically if comparing Figure 7a with Figure 7b.

Comparing the FT-IR spectra of GO and Cu@graphene (Figure 7c), it can be seen that the absorption peaks of both hydroxyl group and carboxyl group are decreased after the plating process, mainly due to the deposition of the copper particles and reduction of GO. The –C=O stretching vibration peak at 1723.97 cm^−1^ disappears for the Cu@graphene sample. Results show that after the GO is reduced, the conjugated structure is restored. The FT-IR spectrum of the graphene (Figure 7c) is smoother than that of the GO (Figure 7a), indicating that the functional groups on the surface of graphene are much less than those on the surface of GO (Figure 7a). Also, the oxygen-containing functional groups on the surface of the GO are decreased [45].

For the FT-IR spectrum of the Ni@graphene (Figure 7d), all the characteristic peaks in the FT-IR spectra of GO are weakened due to the deposition of nickel particles onto the surfaces of graphene sheets. The –C=O stretching peak at 1723.97 cm^−1^ disappears but there are –C–H anti-symmetrical stretching vibration peak at 2922.98 cm^−1^ and –C–H symmetrical stretching vibration peak at 2851.57 cm^−1^, indicating that the –COOH groups on the surfaces of the GO sheets were reduced into –CH_2_OH by using the NaBH_4_. This phenomenon shows that there are new chemical bonds generated between nickel particles and graphene, not just a simple physical adsorption. This chemical bond formations from the reactions can be written using Equations (2) and (3) [44,46]:

(2) $$4{\mathrm{Ni}}_{2}^{2+}+{\mathrm{BH}}_{4}^{-}+8{\mathrm{OH}}^{-}\to 4\mathrm{Ni}+{\mathrm{BO}}_{2}^{-}+6H_{2}O$$

(3) $$-\mathrm{COOH}\overset{\left[{\mathrm{BH}}_{4}^{-}\right]}{\to }-{\mathrm{CH}}_{2}\mathrm{OH}$$

**Figure 8:**
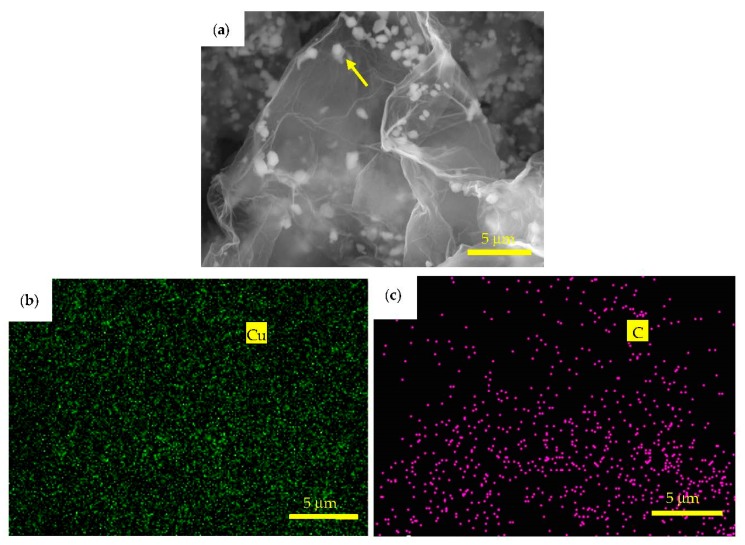
(**a**) Backscattered electron image and (**b**) to (**c**) energy dispersive X-ray spectrometer Cu element mapping and C element mapping of Cu@graphene.

Figure 8a is a back-scattered electron (BSE) image of Cu@graphene synthesized using the in-situ co-reduction method. The white nanoparticles are uniformly dispersed in a semi-transparent and folded graphene substrate and no obvious aggregation is observed. The white fine particles are copper based on the energy dispersive X-ray spectrometer analysis. The images of energy dispersive X-ray spectrometer elemental mapping of the Cu@graphene after the reduction process are shown in Figure 8b,c. Results show that there are elements of C and Cu which are uniformly distributed in the Cu@graphene. The copper particles coated on the graphene tend to be deposited onto the edges and folds of graphene sheets. This is consistent with the reported distribution of copper particles on the surface of graphene in the Cu@graphene in the literature [46].

**Figure 9:**
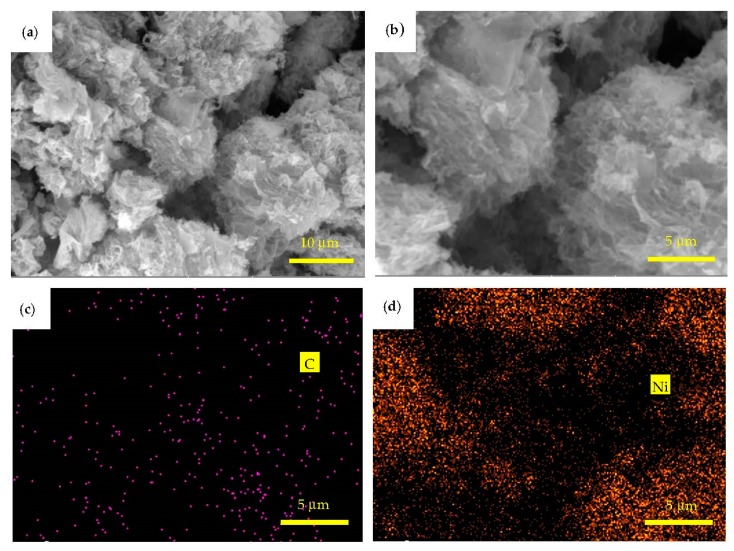
(**a**,**b**) Backscattered electron image and (**c**,**d**) energy dispersive X-ray spectrometer C element mapping and Ni element mapping of Ni@graphene.

Figure 9a is a BSE image of Ni@graphene. It can be seen that the graphene is made up of a wrinkled and folded membrane. From Figure 9b, the graphene sheets are stacked and agglomerated, probably due to the fact that the monolayer nickel-coated graphene sheets are not completely dried before bonded with the graphene layer, which has also been reported in the literature [16]. EDS elemental mapping of the Ni@graphene is shown in Figure 9c,d. Results showed that there are elements of C and Ni uniformly distributed in the graphene.

**Figure 10:**
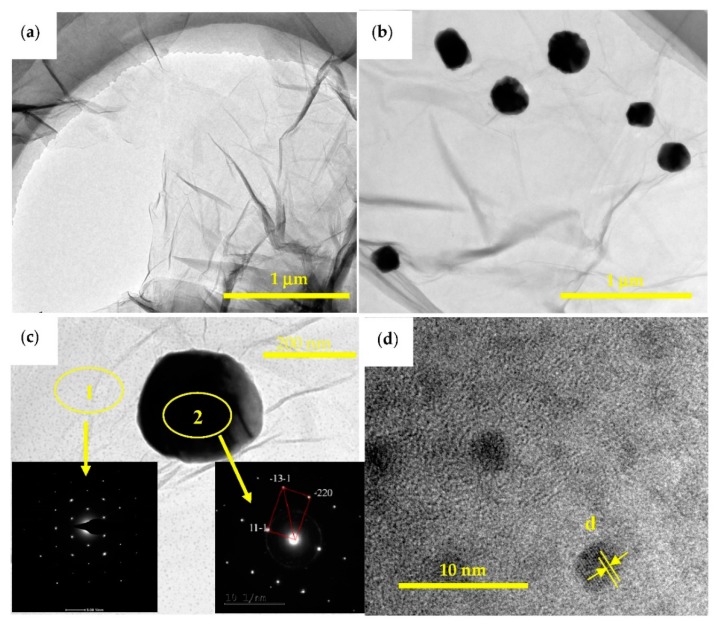
TEM images of Cu@graphene (**a**) original GO (**b**) Cu@graphene after reduced by ascorbic acid; (**c**) single Cu particle on graphene; Insets are the corresponding Selected area diffraction (SAED) patterns; (**d**) high resolution transmission electron microscope of Cu particle on graphene.

TEM images of GO and Cu@graphene are shown in Figure 10. The GO sheets were exfoliated into thin layers and its specific surface areas are quite large as shown in Figure 10a. Formation of large two-dimensional planes of the reduced GO results in a uniform deposition of copper on its surface. At the same time, oxygen-containing functional groups on the surface and edges of GO can react with copper ions, which lead to the uniform distribution of copper on the GO surfaces during plating process. Figure 10b shows that there are black particles with diameters of ~200 nm distributed in the substrate background. Selected area diffraction (SAED) patterns (Figure 10c) confirm that the black area is copper and the gray background area is graphene. Graphene exhibits a yarn-like and translucent layered structure and its surface is not perfectly flat but with a wrinkled and folded sheet-like morphology. This is because the graphene is a two-dimensional material and folds and ripples are easily generated in order to maintain its thermodynamic stability [8]. This is consistent with the microscopic morphologies of graphene reported in the literature [24,47]. The copper particles on the surface of graphene are mainly distributed in the folds and marginal areas of the graphene, which is consistent with what has been reported in the literature [22]. This is mainly due to the larger energy and higher catalytic activity of the folds and marginal areas, which enhances the easier deposition of metal particles. Distinct lattice fringes of Cu can be observed in the high resolution transmission electron microscope image as shown in Figure 10d. The lattice parameter was measured to be 0.21125 nm, corresponding to the interplanar spacing of Cu (111) plane. This indicates the successful obtaining of Cu@graphene, similar to those reported in the literature [46]. These results are also consistent with those from the XRD and SEM.

**Figure 11:**
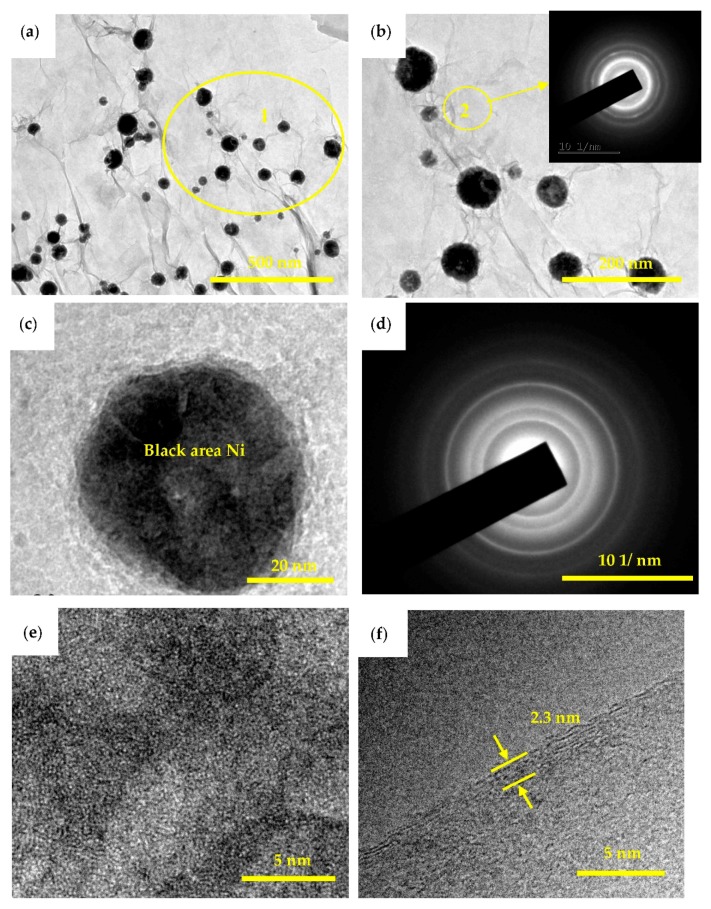
TEM images of Ni@graphene (**a**) and (**b**) TEM picture of Ni@graphene after reduced by sodium ascorbate; Insets are the corresponding SAED patterns; (**c**) single Ni particle on graphene; (**d**) the electron diffraction pattern of (**c**); (**e**) high resolution transmission electron microscope of Ni particle on graphene; (**f**) high resolution transmission electron microscope of graphene.

TEM image of the Ni@graphene is shown in Figure 11a. The SAED pattern taken from the enlarged area of Ni@graphene (Figure 11b) indicates that there are several concentric halo patterns. The boundary of each halo is not clear, which confirms that the gray area is a signal of amorphous carbon, it could be caused by the defects of graphene during the reduction. Figure 11c shows a single Ni particle whose size is around 55 nm and the Ni particle is semi-amorphous or nano-crystalline, as illustrated by the SAED pattern (Figure 11d). This is consistent with XRD results and also those reported in the literature [48,49]. The electroless nickel plating generally produced an amorphous structure and post-annealing was needed for its subsequent conversion into crystalline structures (generally at 701–719 K) [47,50,51,52]. The crystalline structures of Ni deposited on the surface of graphene were reported to be influenced by the post-treatment temperature after plating. When the temperature is less than 473.15 K, the nickel is amorphous; whereas it is crystallized when the temperature is higher than 573.15 K. Nickel oxide can be formed at much higher temperatures [44]. The post-treatment temperature used in this study was 333.15 K, lower than previously reported. Therefore, this is the main reason for widening of the SAED diffraction peak or poor crystallinity of Ni, which is consistent with the result of XRD analysis. The crystal structures of nickel deposited on the surface of graphene may also be affected by the synthesis methods. For example, nickel/graphene prepared using a chemical vapor deposition method produced a crystal structure, [24] and Ni-nanoparticles/graphene composite fabricated using a solvothermal method also showed a crystalline structure [17]. The of the single Ni particle is shown in Figure 11e. Clearly the crystallinity of the deposited nickel is not good, consistent with the results obtained from both electron diffraction and XRD analysis. The distribution of nickel particles (Figure 11a) mainly exists in the edges and folded areas of graphene, similar to those reported in literature [50]. Also, the of graphene is shown in Figure 11f and graphene of ~6 atomic layers can be clearly observed, with a thickness of 2.3 nm, as denoted by the yellow arrow in Figure 11f. Clearly a few layers of graphene in Ni@graphene can be formed using our proposed method, which further confirms the AFM result and analysis.

### 3.2. Cohesive Interfacial Energy Analysis between Graphene and Metal Using First-Principle Calculations

Based on DFT calculation results, the binding energy of Ni atoms adsorbed onto the graphene is 2.078 eV, whereas the binding energy of Ni atoms adsorbed on the graphene with the defect is 3.214 eV. Meanwhile, the binding energy of Cu atoms adsorbed onto the intrinsic graphene is 1.778 eV, whereas that of Cu atoms adsorbed on the graphene with the defect is 5.114 eV. For both cases, the binding of metal atom and graphene is a chemical process, which verifies the experimental results in the previous sections. Meanwhile, results show that the binding energy of atomic adsorption on defect of graphene is larger than that on the intrinsic graphene, which clearly show that the metal atoms are preferably adsorbed on the defects in the graphene. This result is also consistent with the literature [53,54].

**Figure 12:**
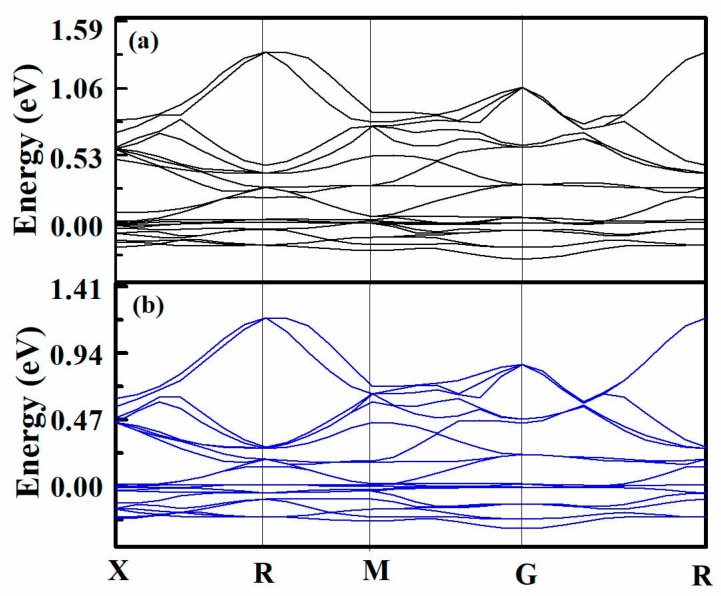
Electronic band structures of (**a**) graphene-Ni and (**b**) graphene-Cu composite systems.

In order to understand the differences of binding strengths between Cu or Ni and graphene, we further analyzed the electronic coupling across the interfaces. The obtained band structures of graphene-Cu(111) system and graphene-Ni(111) system are plotted in Figure 12. The results show that the Fermi level is pinned to the Cu or Ni bands, that is, electrons are transferred from π bands in the graphene layer onto metal bands. The coupling between the orbital of carbon atoms and the orbital of metal atoms is weak and electrons in the metal bands are transferred into the graphene layer. Consequently, the Fermi level is close to the boundary between conjugated π bands of the graphene. The coupling between the *Pz* orbital of carbon atoms and the metal atoms is strong. Therefore, we find that, except for the observed charge transfer between graphene and metal orbitals, a strong coupling is obtained between the band structures of graphene and metal.

### 3.3. Formation Mechanisms of Metal@Graphene

**Figure 13:**
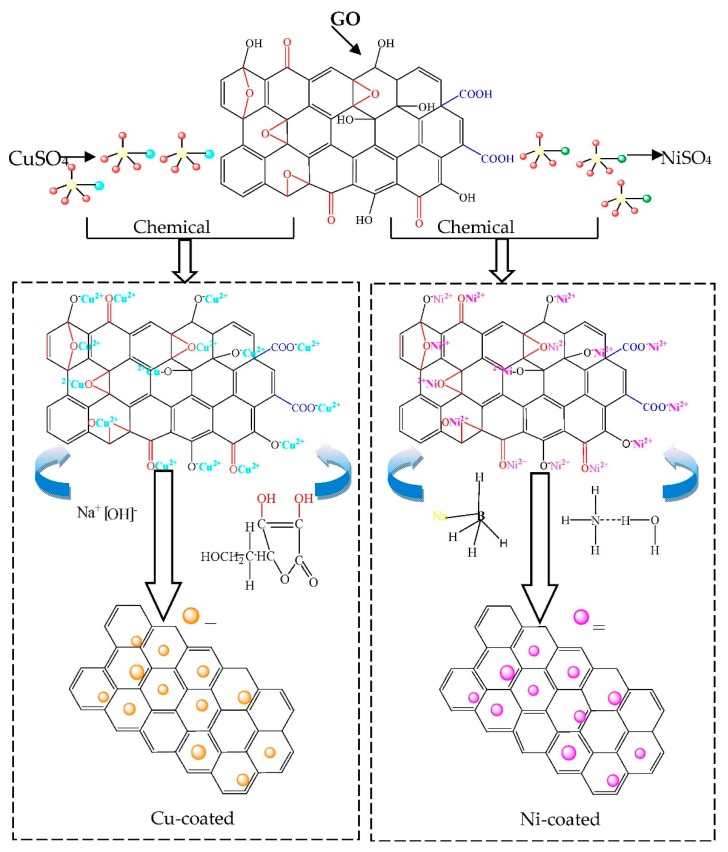
Schematic illustration of reaction mechanism of Cu@graphene and Ni@graphene.

Based on the characterization results, the formation mechanism of metal@graphene is proposed as illustrated in Figure 13. Our in-situ co-reduction method for fabrication of Cu@graphene or Ni@graphene composites starts from the selection of the appropriate salts which can provide suitable metal ions according to the plated metal (i.e., copper sulfate was used for copper plating). Anions of the oxygen-containing functional groups at the edges and surfaces of the GO attract copper ions or nickel ions in the plating solution, therefore, a large amount of copper ions or nickel ions are adsorbed on the surfaces and edges of the GO.

In the second stage, a suitable chemical reagent and a strong reducing agent (i.e., ascorbic acid in the copper plating and sodium borohydride in the nickel plating) are applied depending on the type of metal to be plated and the chemical reduction processes to be initiated.

The third step is the most important one which is in-situ and co-reaction process. The copper ions or nickel ions adsorbed on the edge and surfaces of graphene oxide are strongly reduced in the alkaline conditions to obtain metal particles and the specific reactions for copper plating are written in Equations (4)–(7). Those of the nickel plating are written in Equation (8):

(4) $${\mathrm{Cu}}^{2+}+2{\mathrm{OH}}^{-}=\mathrm{Cu}{\left(\mathrm{OH}\right)}_{2}$$

(5) $$\mathrm{Cu}{\left(\mathrm{OH}\right)}_{2}=\mathrm{CuO}+H_{2}O$$

(6) $$\mathrm{Cu}{\left(\mathrm{OH}\right)}_{2}+C_{6}H_{8}O_{6}\to {\mathrm{Cu}}_{2}O+C_{6}H_{6}O_{6}+H_{2}O$$

(7) $${\mathrm{Cu}}_{2}O+C_{6}H_{8}O_{6}\to C_{6}H_{6}O_{6}+H_{2}O$$

(8) $$4{\mathrm{Ni}}^{2+}+{{\mathrm{BH}}_{4}}^{-}+8{\mathrm{OH}}^{-}\to 4\mathrm{Ni}+{{\mathrm{BO}}_{2}}^{-}+6H_{2}O$$

Simultaneously, GO is reduced by the strong reducing agent of ascorbic acid or sodium borohydride to produce a graphene layer. The –COOH functional group on the surface of the GO is reduced to –CH_2_OH with the reduction actions of ascorbic acid or sodium borohydride. The reduction reactions can be verified from our FT-IR analysis. The reactions that occur in this process can be expressed as follows:

(9) $$-\mathrm{COOH}\overset{C_{6}H_{8}O_{6}}{\to }-{\mathrm{CH}}_{2}\mathrm{OH}$$

(10) $$-\mathrm{COOH}\overset{\left[{\mathrm{BH}}_{4}^{-}\right]}{\to }-{\mathrm{CH}}_{2}\mathrm{OH}$$

Since the newly formed graphene has a large surface energy, the metal ions in the solution are easily adsorbed onto the surface of the graphene and then deposited onto its surface and the inherent “defects” of the graphene cause the metal ions to form a good chemical bonding [55]. This has been verified by both the Infrared and Raman analysis results.

## 4. Conclusions

In this work, GO of 2~3 atomic layers were obtained using the Hummer’s method. Using GO, copper sulfate and ascorbic acids (or nickel sulfate and sodium borohydride), the Cu@graphene powders (or Ni@graphene powders) with a homogeneous distribution of metal nanoparticles on the surface of graphene were obtained using an efficient in-situ co-reduction method. The formation mechanism of metal@graphene can be explained: for example, the metal ions and graphene firstly form a chemical bond and the GO and the metal ions are simultaneously reduced by the reducing agents to finally obtain the metal@graphene. The binding of metal atom and graphene is a chemical process which can be verified based on the results from the first-principle calculations.

## Figures and Tables

**Table 1:** The compositions of metal plating solution (Cu or Ni) and reaction conditions.

Plating Solution	Chemical	Function	Concentration
Copper	CuSO_4_·5H_2_O	Source Cu	50 g·L^−1^
	PVP	surfactant agent	0.4 mg·mL^−1^
	Ascorbic	Reducing agent	2 g·L^−1^
	NaOH	Adjust the pH value	50 g·L^−1^
Nickel	NiSO_4_·6H_2_O	Source Ni	1.3 g·L^−1^
	PVP	surfactant agent	0.4 mg·mL^−1^
	NaBH_4_	Reducing agent	8 g·L^−1^
	NH_3_·H_2_O	Adjust the pH value	25–28 wt%
